# Platelet derived TGF-β promotes cervical carcinoma cell growth by suppressing KLF6 expression

**DOI:** 10.18632/oncotarget.19912

**Published:** 2017-08-03

**Authors:** Ao-Di He, Shao-Ping Wang, Wen Xie, Wei Song, Shuo Miao, Ru-Ping Yang, Ying Zhu, Ji-Zhou Xiang, Zhang-Yin Ming

**Affiliations:** ^1^ Department of Pharmacology, School of Basic Medicine, Tongji Medical College of Huazhong University of Science & Technology, Wuhan 430030, China; ^2^ Department of Hepatobiliary Surgery, Affiliated Futian Hospital of Guangdong Medical College, Shenzhen 518033, China; ^3^ The Key Laboratory for Drug Target Researches and Pharmacodynamic Evaluation of Hubei Province, Wuhan 430030, China

**Keywords:** platelet, platelet releasate, HeLa cervical carcinoma cells, Krüppel-like factor 6, transforming growth factor beta

## Abstract

Platelets in the primary tumor microenvironment play crucial roles in regulating tumor growth, metastasis, and angiogenesis, but the underlying mechanisms are unclear. Here, we show that platelet releasates exhibited a proliferative effect on HeLa cells, and this effect correlated with a reduction of KLF6 expression. After incubation with either washed human platelets or collagen-related peptide (CRP) activated platelet releasates, expression of KLF6 in the HeLa cervical tumor cell line was markedly reduced. However, no significant difference was observed between control HeLa cells and HeLa cells incubated with resuspended activated platelet pellet. Moreover, the platelets’ promoting effect on HeLa cell growth was significantly abolished in KLF6 silenced HeLa cells. In addition, blocking TGF-β signaling with SB431542, a TGF-β receptor inhibitor, also counteracted the effect of platelets on proliferation and KLF6 expression in HeLa cells. From these findings, we conclude that platelet derived TGF-β promotes proliferation of HeLa cells by decreasing the expression of KLF6. The discovery that KLF6 is a key target of platelet-derived TGF-β signaling in HeLa cells identifies a potential new therapeutic target for the prevention and treatment of cervical carcinoma.

## INTRODUCTION

Cervical carcinoma is a worldwide disease and the second prevalent common cancer in women which constitutes a significant public health problem.[1, 2]. Because of the sharply increasing incidence of cervical cancer [3], a detailed understanding of the molecular mechanisms associated with cervical carcinoma is needed to improve our approaches to treatment of this disease.

The relationship between platelets and cancer has been recognized for more than one hundred years, since the proposal of Trousseau syndrome in 1865 [4]. Extensive experimental evidences have been generated in support of an important role for circulating platelet in cancer progression, and researches revealed a role for physiologic platelet receptors and platelet granule contents in cancer growth, dissemination and angiogenesis [5–8]. Additionally, it has been reported that platelets accelerated the metastasis of cervical carcinoma by GPIIb/IIIa and α_v_β3 integrins [9]. However, the effects of platelet on cervical cancer cell proliferation and the molecular mechanisms underlying these associations have not been fully explored.

Transforming growth factor beta (TGF-β) controls the proliferation and differentiation of many types of non-malignant cells and is necessary for tumor cell extravasation and metastasis formation [10]. Platelets are a major source of TGF-β[11]. It has been reported that TGF-β1 secreted from platelets promotes the proliferation of ovarian cancer cells [12], indicating a potential role for TGF-β in platelet and cancer cell interactions. Although previous studies showed that HeLa cells treated with TGF-β1 for 24 hour resulted in an increasing growth [13], whether platelet-derived TGF-β involved in platelet- Hela cell interaction is still unknown.

Krüppel-like factor 6 (KLF6) is a ubiquitously expressed zinc finger transcription factor and has been characterized as a tumor suppressor gene that mediates growth suppression in a variety of human cancers [14–16]. Research has shown that TGF-β can enhance the cooperation between KLF6 and Sp1 to regulate target genes in cells including HeLa cell [17]. Therefore, we hypothesized that the pro-proliferative effects of platelets on tumor cells are attributed to the ability of platelet-derived TGF-β to decrease the expression of KLF6 in tumor cells.

In the present study, we found that platelets or platelet granule contents, which are released upon platelet activation, reduced the expression of KLF6 and promoted growth in HeLa cells. Knockdown of KLF6 expression with siRNA substantially attenuated the pro-proliferative effect of platelets. Additionally, blocking TGF-β signaling with a TGF-β receptor inhibitor abrogated the stimulatory effect of platelets on HeLa cells. Taken together, these findings suggest that platelet releasates, especially TGF-β, promote the proliferation of HeLa cells by decreasing expression of KLF6.

## RESULTS

### Platelets promote the growth of HeLa cells via reduced KLF6 expression

**Figure 1:**
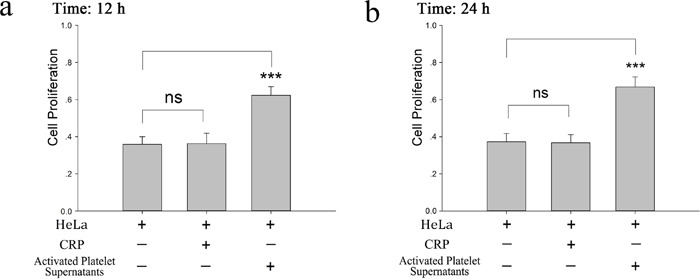
Proliferative effect of platelet releasates on HeLa cells **(a-b)** Washed platelets (2×10^8^/ml) were treated with CRP (0.8 μg/ml). When platelets were fully activated, supernatants were isolated by centrifugation at 1000×g for 10 min and then incubated with HeLa cells. MTT assays were performed to determine the proliferation effects of releasates from activated platelets on HeLa cells at 12 hour (a) or 24 hour (b). HeLa cells cultured in fresh medium were compared as a control. The results are expressed as the mean ± SEM (^***^p<0.001 compared with control). Five duplicate wells were set up for each group, and the experiment was repeated ten times.

**Figure 2:**
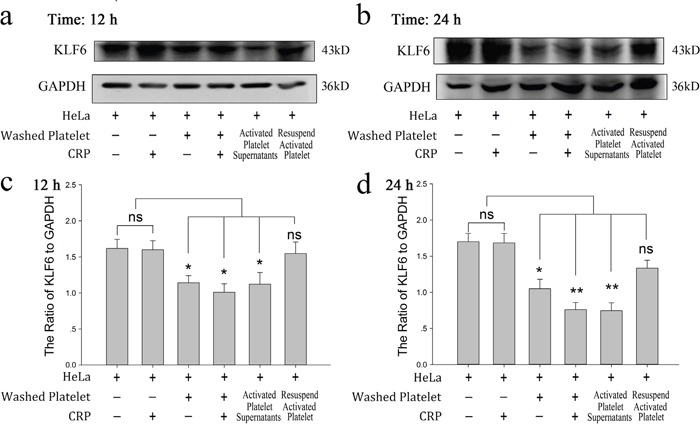
Effect of platelets on HeLa cells via reduced KLF6 expression **(a-b)** HeLa cells were incubated with resting platelets, platelets treated withspecific stimulator CRP (0.8 μg/ml), washed platelets, washed platelets plus CRP, releasates of CRP-activated platelets, and the resuspended CRP-activated platelets pellet. KLF6 expression was measured by Western blot analysis in HeLa cells after a 12-hour (a) or 24-hour (b) treatment. **(c-d)** Quantitative analysis of the ratio of KLF6 levels to GAPDH is shown; data are presented as the mean ± SEM (n = 8, ^*^p<0.05 and ^**^p<0.01 compared with control).

In this study, we investigated the influence of platelets on HeLa cell proliferation. As shown in Figure 1a and 1b, CRP (0.8 μg/ml)-activated platelet supernatants accelerated the proliferation of HeLa cells in the MTT assay at 12 and 24 hour. In accordance with this promoting effect, KLF6, a tumor suppressor gene expressed in HeLa cells and deficient in platelets (Supplementary Figure 1), was proposed to play a potential role. In Figure 2a and 2b, after the incubation of HeLa cells with platelets treated with or without CRP for 12 and 24 hour, the expression of KLF6 in both groups was significantly suppressed compared with the HeLa control group. To define whether the platelet itself or its secretions were involved in this effect, the releasate in the supernatant was separated from the activated platelets by centrifugation. The supernatants also had a suppressive effect on KLF6 expression in HeLa cells. Conversely, the platelet itself, which was exhausted with platelet releasates and resuspended in fresh medium showed no significant influence on KLF6 expression (Figure 2a–2d).

### KLF6 gene silence dampened the proliferative effect of platelets on HeLa cells

**Figure 3:**
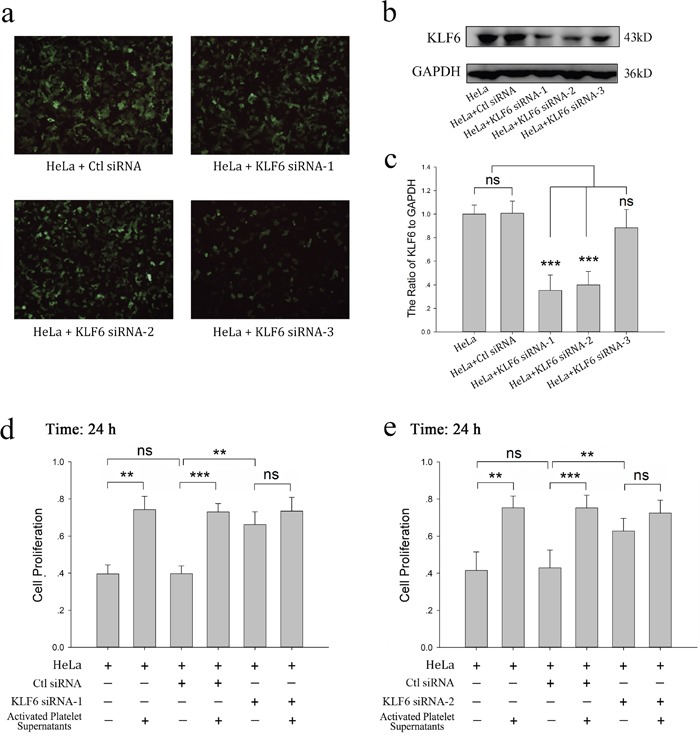
KLF6 gene silence dampened the proliferative effect of platelets on HeLa cells **(a-c)** HeLa cells grown on 6-well plates were transfected with either control siRNA (Ctl siRNA) or 3 different KLF6 siRNAs (KLF6 siRNA-1, KLF6 siRNA-2 and KLF6 siRNA-3) for 48 hour. The expression of green fluorescence protein (GFP) was measured by a fluorescence microscope with 100X magnification (a). Four days after transfection, KLF6 expression was visualized by Western blot analysis (b). GAPDH was used as a loading control. (c) The ratio of KLF6 level to GAPDH summarized as a bar graph, data are presented as the mean ± SEM (n = 4, ^***^p<0.001 compared with control). **(d-e)** Normal HeLa cells, HeLa cells transfected with control vector, KLF6 siRNA-1 (d) or KLF6 siRNA-2 (e) were incubated with CRP (0.8 μg/ml)-activated platelet releasates. The MTT assay was performed to determine cell proliferation after a 24-hour incubation, and cells cultured in fresh medium were compared as control. Data are presented as the mean ± SEM (^**^p<0.01, ^***^p<0.001 compared with controls). Five duplicate wells were set up for each group, and the experiment was repeated six times.

To confirm the critical role of KLF6 in the platelet-mediated pro-proliferative effects on HeLa cells, HeLa cells were transfected with siRNA for KLF6 to knockdown its expression. As shown in Figure 3a–3c, siRNA1 and siRNA2 decreased the expression of KLF6 in HeLa cells by 64.7% and 60.0%, respectively. Neither siRNA3 nor scrambled siRNA significantly affected KLF6 expression, though the transfection efficiency for all four siRNAs was similar. Compared with scrambled siRNA-transfected HeLa cells, both siRNA1- and siRNA2- transfected HeLa cells had an improved growth rate, correlating with the reduced level of KLF6 expression (Figure 3d and 3e). Though platelet releasates were able to promote the growth of HeLa cells transfected with scrambled RNA, they had no effect on the proliferation of HeLa cells in which KLF6 was silenced by either siRNA1 or siRNA2 (66.27% vs 73.46% and 62.77% vs 72.47%, respectively; p>0.05) (Figure 3d and 3e).

### Platelet-derived TGF-β promotes the proliferation of tumor cells

**Figure 4:**
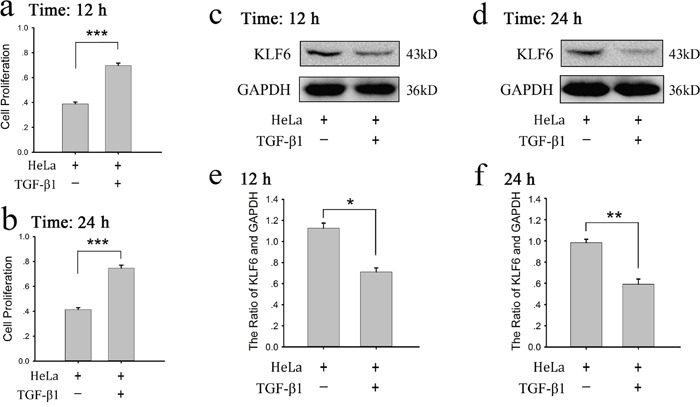
Recombinant TGF-β1 increased proliferation and decreased KLF6 expression of HeLa cells **(a-b)** HeLa cells were treated with human recombinant TGF-β1 (10 ng/ml) for 12 (a) or 24 (b) hours, the proliferation of cells was assessed by MTT assay. Cells cultured in fresh medium were compared as a control. The results are expressed as the mean ± SEM (^***^p<0.001 compared with control). Five duplicate wells were set up for each group, and the experiment was repeated 3 times. **(c-f)** HeLa cells were treated with TGF-β1 (10 ng/ml) for 12 (c) or 24 (d) hours, expression of KLF6 was measured by a western blot analysis. Cells cultured in fresh medium were used as a control. A quantitative analysis of the ratio of KLF6 to GAPDH expression is shown (e-f), and data are presented as the mean ± SEM (n = 3, ^*^p<0.05, ^**^p<0.01 compared with control).

Above study showed platelet granule contents promote HeLa cell growth and attenuate KLF6 expression of HeLa cells. Platelets are an important source of bioavailable TGF-β for tumor cells [18]. Reduced expression of TGF-β1 receptors was found along with a decreased proliferation of ovarian cancer cells when they were exposed to platelets [12]. Therefore we proposed that platelet-derived TGF-β was involved in tumor growth and affected KLF6 expression. We firstly detected whether human recombinant TGF-β1 could affect proliferation and KLF6 expression of HeLa cells. Figure 4 showed that TGF-β1 could markedly increase HeLa cell growth (Figure 4a and 4b) and reduce expression of KLF6 in HeLa cells (Figure 4c–4f) at the 12 and 24 hour time points.

**Figure 5:**
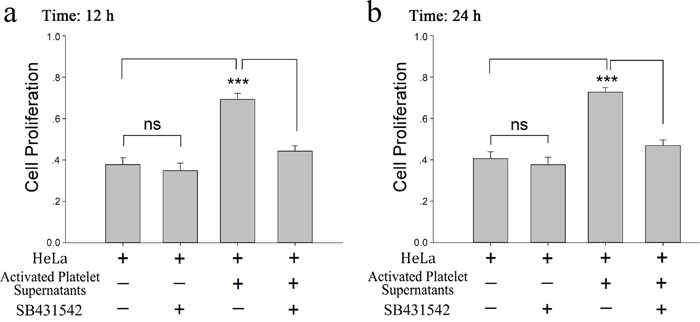
Platelet derived TGF-β promotes HeLa cell proliferation **(a-b)** Supernatants from CRP (0.8 μg/ml)-activated platelets (2×10^8^/ml) were isolated by centrifugation at 1000×g for 10 min. HeLa cells were treated with the platelet supernatants in the absence and presence of the TGF-β receptor inhibitor SB431542 (10μM). The MTT assay was used to determine the proliferation effects of the releasates from activated platelets on HeLa cells at 12 hour (a) or 24 hour (b). HeLa cells cultured in fresh medium were used as control. Each experiment was repeated 10 times in triplicate. Data are presented as the mean ± SEM (n = 10, ^***^p<0.001 compared with controls).

**Figure 6:**
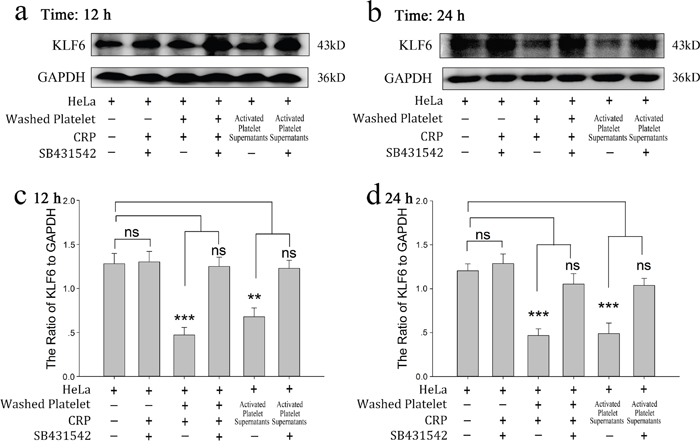
Platelet-derived TGF-β regulates the expression of KLF6 in cervical carcinoma cells **(a-b)** HeLa cells were incubated with the TGF-β receptor inhibitor SB431542 (10 μM), platelets treated with CRP (0.8 μg/ml), washed platelet plus CRP, and supernatants of CRP-activated platelets in the absence and presence of SB431542 (10 μM). KLF6 expression was detected by Western blot analysis in HeLa cells after 12 hour (a) or 24 hour (b) of treatment. **(c-d)** Quantification of data are presented as the mean ± SEM (n = 5, ^**^p<0.01, ^***^p<0.001 compared with controls).

To demonstrate the role of platelet-derived TGF-β in HeLa cell growth, SB431542, a TGF-β receptor blocker, was incubated with HeLa cells before treated with platelet or its releasate. The promoting effect of platelet secretion on the proliferation of HeLa cells was reversed by SB431542 (Figure 5a and 5b). Both CRP (0.8 μg/ml)-treated platelets and their releasate could reduce the expression of KLF6 at the 12 and 24 hour time points (Figure 6a and 6b), which promoted proliferation of HeLa cells. These effects could be blocked by the TGF-β receptor inhibitor (10 μM) (Figure 6a–6d).

## DISCUSSION

It has been well recognized that platelet and its releasates are key players in the regulation of tumor angiogenesis and metastasis [19]. In support of this concept, we found that the inclusion of washed human platelets in culture media significantly promoted HeLa cell proliferation. Surprisingly, we found that the expression of KLF6 was reduced by incubating HeLa cells with platelets. A TGF-β receptor antagonist reversed the effects on HeLa cell proliferation and KLF6 expression.

KLF6 is a ubiquitously expressed tumor suppressor gene. In cervical carcinoma, loss and/or mutation of KLF6 results in its inactivation and/or downregulation, contributing to cervical cancer pathogenesis via the effects on targeted genes that control cell proliferation and differentiation [20, 21]. In our experiments, HeLa cells incubated with platelet releasates exhibited lower levels of expression of KLF6 and enhanced proliferation relative to HeLa cells cultured in the absence of platelet pellet. This observed effect of platelets on KLF6 expression was independent of direct cross contact between platelet and tumor cells because the pellet of CRP-activated platelets, which had secreted all of its granules components, had no effect on the expression of KLF6 in HeLa cells. In addition, the proliferation of HeLa cells in which KLF6 was silenced showed no significant difference in the presence of platelet releasate. These data demonstrate that the pro-proliferative effect of platelet releasates on HeLa cells is due to the downregulation of KLF6 expression in HeLa cells by factors released from platelets and is independent of platelet-tumor cell direct interaction.

TGF-β has been reported to be involved in the regulation of KFL6 expression in different cells [17, 22], and recombinant TGF-β1 could increase cell proliferation and reduce KLF6 expression in HeLa cell line. Moreover, recent studies showed that platelet-derived TGF-β is known to be involved in the EMT and metastasis of tumor cells via the TGF-β/Smad and NF-kB pathways [23]. We therefore hypothesized that platelet-derived TGF-β is responsible for the effects of platelet releasates on KLF6 expression and HeLa cell proliferation. Consistent with our hypothesis, blocking TGF-β signaling with a TGF-β receptor inhibitor abolished the platelet releasate-induced downregulation of KLF6 expression and the proliferation of HeLa cells.

Collectively, these data reveal a pivotal role for KLF6 in the contribution of platelets and their releasates such as TGF-β to the proliferation of HeLa cells. These findings broaden our knowledge about the role of platelets in tumor progression, which may offer a novel treatment strategy in cervical carcinoma.

## MATERIALS AND METHODS

### Tumor cell lines and transfection

HeLa cells were obtained from the Chinese Center for Type Culture Collection (CCTCC). Cells were routinely cultured in Dulbecco's modified Eagles' medium (DMEM, HyClone Logan, UK) supplemented with 10% newborn calf serum (Gibco, Grand Island, NY, USA) and antibiotics (penicillin, streptomycin 100 g/mL, Amresco, OH, USA) and incubated in a humidified incubator containing 5% CO_2_, at 37°C. siRNA against KLF6 and control non-targeting siRNA were obtained from Genechem (Shanghai, China). Cells were seeded overnight in six-well plates at a density of 5×10^5^ cells per well. Cells were transfected with the siRNA targeting KLF6 or the control siRNA using polybrene (10 μg/ml, Sigma-Aldrich, St. Louis, MO, USA), according to the manufacturer's instructions. Transfection efficiency was determined by green fluorescent protein (GFP) expression using fluorescence microscopy. When the GFP expression was greater than 70% in each group after transfection, Western blot analysis was performed to determine which siRNA sequence sufficiently suppressed KLF6 expression.

### Treatment of tumor cells with washed human platelets

Human blood was collected from healthy volunteers. Washed platelets were prepared as described previously [24] and resuspended in fresh medium. When incubated with HeLa cells, platelets were activated by collagen-related peptide (CRP, 0.8 μg/ml), which was obtained as a gift from the Blood Research Institute at the Blood Center of Wisconsin. Platelet releasates were collected following centrifugation of CRP-stimulated platelets at 1000×g for 10 min.

Before being incubated with platelets or platelet releasates, HeLa cells were seeded in six-well plates and incubated at 37°C in a humidified incubator containing 5% CO_2_. When HeLa cells reached approximately 80% confluence, washed human platelets (2×10^8^/ml), platelet releasates or platelet pellets were added. In some experiments, HeLa cells were treated with human recombinant TGF-β1 (10 ng/ml, Sigma-Aldrich, St. Louis, MO, US) or TGF-β1 receptor inhibitor, SB431542 (10 μM, Selleckchem, Houston, TX, USA), before incubation with human platelets.

All experimental procedures were approved by the Ethics Committee for the Use of Human Subjects of Huazhong University of Science and Technology. These studies were conducted in accordance with the Declaration of Helsinki.

### Western blot

After treatment, cells were washed with PBS. Lysis of cells was performed on ice by scraping in lysis buffer supplemented with 2% protease inhibitors (Calbiochem, MA, USA). Proteins present in cell lysates were separated by SDS-PAGE and transferred to PVDF membranes (EMD Millipore Corporation, Billerica, MA, USA). The membranes were incubated with a primary mouse antibody against KLF6 (1:250, Santa Cruz Biotechnology, CA, USA). A horseradish peroxidase-conjugated anti-mouse antibody was used as a secondary antibody, and signals were detected by enhanced chemiluminescence (Thermo Scientific, USA). GAPDH (1:5,000, Cell Signaling Technology, MA, USA) was used as a protein loading control.

### MTT assay

The effect of platelets on cell proliferation was determined with a colorimetric 3-(4, 5-dimethylthiazol)-2, 5-diphenyltetrazolium bromide (MTT) (Amresco, OH, USA) assay. HeLa cells were plated at an initial density of 4000 cells per well into 96-well plates and incubated at 37°C overnight. The cells were then incubated with platelet releasates. Negative controls were treated with medium only. The absorbance (OD) was measured with a microplate reader (Tecan Sunrise, Grodig, Austria) at a 490 nm wavelength.

### Statistical analysis

Data throughout the manuscript are presented as the mean ± SEM. Comparisons were made using Student's t-test, with p<0.05 considered statistically significant.

## SUPPLEMENTARY MATERIALS FIGURE



## Abbreviations

CRP: collagen-related peptide
KLF6: Krüppel-like factor 6
MTT: 3-(4, 5-dimethylthiazol)-2, 5-diphenyltetrazolium bromide
siRNA: small interfering RNA
TGF-β: transforming growth factor beta
