# Supplementary material for: Platelet derived TGF-β promotes cervical carcinoma cell growth by suppressing KLF6 expression
Source: Oncotarget. 2017 Aug 3;8(50):87174–81. doi: 10.18632/oncotarget.19912 (PMC5675624; doi:10.18632/oncotarget.19912)
Supplement: Supplementary file 1 [file oncotarget-08-87174-s001.pdf]

Platelet derived TGF-β promotes cervical carcinoma cell growth by suppressing KLF6 expression

SUPPLEMENTARY MATERIALS

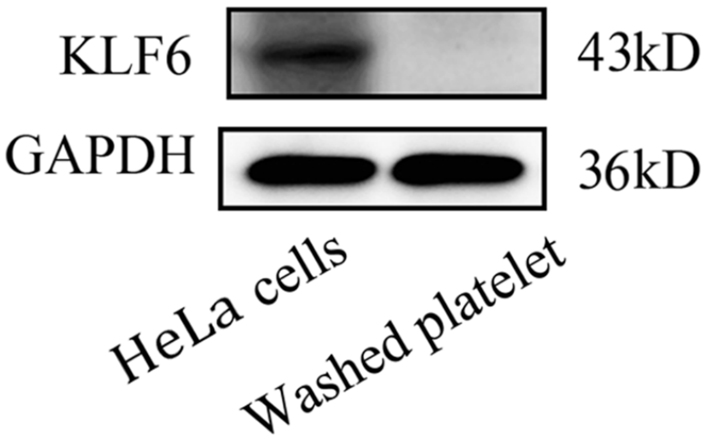

Supplementary Figure 1: Expression of KLF6 in HeLa cells and platelets. Western blot analysis of KLF6 expression in HeLa cells and washed platelets. GAPDH served as a loading control.
